# Risk Assessment and Management Program (RAMP) on knee osteoarthritis in primary care—a one-year pragmatic randomized controlled trial

**DOI:** 10.1186/s13063-026-09469-x

**Published:** 2026-02-03

**Authors:** Regina Wing-Shan Sit, Benjamin Hon-kei Yip, Shirley Yue-Kwan Choi, Martin Chi-sang Wong, Sheung-wai Law, Chor Yin Lam, David Hunter, Samuel Yeung-Shan Wong

**Affiliations:** 1 The Jockey Club School of Public Health and Primary Care, The Chinese University of Hong Kong, Sha Tin, New Territories Hong Kong; 2 Department of Family Medicine, New Territories East Cluster, Hospital Authority, Sha Tin, New Territories Hong Kong; 3 Department of Orthopaedics & Traumatology, The Chinese University of Hong Kong, Sha Tin, Hong Kong; 4 Department of Orthopaedics and Traumatology, School of Clinical Medicine, The University of Hong Kong, Sha Tin, Hong Kong; 5 Sydney Musculoskeletal Health, Kolling Institute, University of Sydney, Sydney, NSW Australia; 6 Rheumatology Department, Royal North Shore Hospital, Sydney, NSW Australia

**Keywords:** Risk Assessment and Management Program (RAMP), Knee osteoarthritis, Primary care

## Abstract

**Background:**

Knee osteoarthritis (OA) is the most common chronic arthritis and is a leading cause of pain and disability. Chronic care model (CCM) has been proved successful in Hong Kong primary care setting. This study aims to assess the clinical effectiveness of a CCM, named Assessment and Management Program on Knee Osteoarthritis (RAMP-Knee OA), compared to usual care in adults with knee OA.

**Methods:**

The study is a 52-week, two-arm, parallel, open-label randomized clinical trial, evaluating the clinical efficacy of RAMP-Knee OA (*N* = 114) versus usual care (*N* = 114) on self-reported knee pain and other secondary outcomes. Measurement instruments will be tested at baseline, 16, 32, and 52 weeks. The primary outcome will be the Western Ontario and McMaster Universities Osteoarthritis Index (WOMAC; 5-point Likert) pain at 52 weeks. Secondary outcomes include a set of biopsychosocial parameters: Physical function will be measured subjectively by WOMAC function subscale and objectively by the 30-second chair and stand performance test. Lower limb muscle mass will be measured by bioimpedance analysis. Physical activity level will be measured by the Chinese International Physical Activity Questionnaire (Short form). Self-management efficacy will be measured by Pain-Self Efficacy questionnaire. Generalized Anxiety Disorder-7 (GAD-7) and Patient Health Questionnaire-9 (PHQ-9) will be used to measure anxiety and depression, respectively. Insomnia will be measured by the 7-item Insomnia Severity Index (ISI), and loneliness will be measured by the 6-item De Jong Gierveld Loneliness Scale. EuroQuol-5D questionnaire will be used to measure health-related quality of life. Both primary and secondary outcomes at different points will be conducted using analysis of covariance, adjusting for baseline values. Secondary analyses include adjustments for potential confounders and exploration of interaction effects of treatment and the potential moderators.

**Discussion:**

The findings will address the evidence-to-practice gap for the implementation of a CCM that incorporates a comprehensive risk assessment, care protocol, self-management support, and scheduled health assessments in Hong Kong.

**Trial registration:**

ClinicalTrials.gov, 1 NCT06283147. Registered on 22 February 2024.

**Supplementary Information:**

The online version contains supplementary material available at 10.1186/s13063-026-09469-x.

## Structured summary {1b}

**Table:** 

Item	Description
Primary Registry and Trial Identifying Number {4}	Registered on clinicaltrials.gov (NCT06283147) on 22/2/2024
Secondary Identifying Numbers	N/A
Source(s) of Monetary or Material Support	Health and Medical Research Fund, Health Bureau, HKSAR
Primary Sponsor and contact information {3b}	N/A
Role of sponsor and funder {3c}	The funder's role is solely to provide monetary support for the project.
Contact for Public Queries	+ 852 2609 5050
Contact for Scientific Queries	+ 852 2609 5050
Public Title	Risk Assessment and Management Program (RAMP) on Knee Osteoarthritis in Primary Care– a one-year pragmatic randomized controlled trial
Scientific title	Risk Assessment and Management Program (RAMP) on Knee Osteoarthritis in Primary Care– a one-year pragmatic randomized controlled trial
Countries of Recruitment	Hong Kong
Health Condition(s) or Problem(s) Studied	Knee Osteoarthritis
Intervention(s)	Risk assessment and Management program
Key Inclusion and Exclusion Criteria	Inclusion criteria include: Age 45 or above, Knee pain for > 3 months, diagnosed with knee osteoarthritis. Exclusion criteria include: potentially serious knee pathology, previous knee replacement, or scheduled replacement within 1 year
Study Type	The study is a two-arm, parallel, doubled-blinded randomised clinical trial
Date of First Enrollment (planned)	8/4/2024
Sample Size	228
Primary outcome(s)	Western Ontario and McMaster Universities Osteoarthritis Index (WOMAC; 5-point Likert) pain score at 52 weeks.
Key Secondary outcome(s)	Physical Function, Lower limb muscle mass, physical activity level, self-management efficacy, anxiety, depression, insomnia, loneliness, health-related quality of life
Ethics Review	This study is approved by the Joint Chinese University of Hong Kong New Territories East Cluster Clinical Research Ethics Committee (Joint CUHK NTEC-CREC). (Reference number: 2023.530)
Individual Trial Participant Data sharing statement	Not intending to share

## Protocol version {2}

Protocol version 1, dated 11th October 2023.

## Introduction

### Background and rationale {9a}

#### Burden of knee osteoarthritis

Knee osteoarthritis (OA) is the most common form of chronic arthritis and a leading cause of pain and disability worldwide. According to the Global Burden of Disease (GBD) 2017 study, the global age-standardized point prevalence and annual incidence rate of OA in 2017 were 3754.2 and 181.2 per 100,000, respectively [1]. Knee OA is age-related [2]; by age 60, the prevalence of knee OA increases to about 10% in both Caucasian and Chinese populations [3, 4]. Individuals with knee OA suffer from greater degrees of pain, activity limitations, psychological distress, and markedly reduced quality of life [5, 6]. Overall, the burden of OA on individuals is best captured in terms of its effect on their quality of life; the global age-standardized years lost due to disability rate in 2017 was 118.8, an increase of 9.6% from 1990 [1]. Despite its clinical burden and socioeconomic costs, there are currently no approved disease-modifying drugs for OA, and symptom palliation is the only alternative [7], with joint replacement reserved for end-stage OA [8, 9].

#### Management of knee OA in primary care-evidence-to-practice gaps

Knee OA is commonly managed in primary care [10, 11]. A systematic review has highlighted that primary care physicians (PCPs) are hampered in their treatment of OA for 2 major reasons: first, by the lack of knowledge of non-surgical management options, and second, by limited access to services that support the core recommended options such as lifestyle and behavioral changes [12]. To address the first gap, the Health Bureau of the Hong Kong Special Administrative Region has published a Reference Framework for Common Musculoskeletal Problems in Primary Care Settings in December 2022, which includes a core document and a module on knee osteoarthritis [13]. The framework is constructed by a panel of local experts with an aim to provide a common clinical reference for PCPs and other allied health professionals, to guide and to facilitate evidence-based practice in primary care. However, no effective strategy is available to deal with the second gap on the access of sustainable support networks for multi-disciplinary input and lifestyle counselling, thus limiting the implementation of core recommended treatments for knee OA.

#### The need of chronic care model for knee OA in Hong Kong primary care

In Hong Kong, the Hospital Authority provides 65% public primary care, running a total of 73 General Out-patient Clinics in Kowloon, Hong Kong Island, and the New Territories, responsible for almost 90% of chronic disease management. Chronic care model (CCM) is a guide to improve healthcare practice and health outcomes for chronic diseases in primary care [14]. Successful CCMs in Hong Kong primary care include the Risk Assessment and Management Program (RAMP) on Diabetes and Hypertension, with favorable long-term clinical effectiveness [15, 16], cost-effectiveness [17], and improved quality of life [18].

To facilitate the implementation of the Reference Framework for knee OA, a theory-informed CCM that incorporates a comprehensive risk assessment, care protocol, self-management support, and scheduled health assessments is going to address the evidence-to-practice gap.

### Explanation for the choice of comparator {9b}

In this research design, the intervention group is compared with a usual-care group. Usual care includes all standard treatments available to patients with knee OA in Hong Kong, as well as any private treatments they seek. As noted, knee OA is commonly managed in primary care, and in Hong Kong, the Hospital Authority provides 65% of public primary care services. The comparator group can mimic the current treatment environment in Hong Kong, and positive results generated from this study can highlight the effectiveness of implementing this CCM in the Hong Kong primary care setting.

### Objectives {10}

Our primary aim is to determine the clinical effectiveness of a CCM named Risk Assessment and Management Program on Knee OA (RAMP-Knee OA) versus usual care in self-reported knee pain in adults with knee OA at 52 weeks. The hypothesis is that participants in the RAMP-Knee OA group will report greater improvement in the Western Ontario and McMaster Universities Osteoarthritis Index (WOMAC) pain subscale, the gold-standard of self-reported outcome in knee OA trials [19].

Our secondary aims are to assess the clinical effectiveness of RAMP-Knee OA versus usual care in terms of self-reported and objectively assessed functional improvement, self-management efficacy, lower limb muscle mass, psychosocial distress, and quality of life at 52 weeks. We hypothesized that participants in the RAMP-Knee OA group will report greater improvement in physical function, self-management efficacy, lower limb muscle mass, psychosocial health, and quality of life.

## Methods: patient and public involvement and trial design

### Patient and public involvement {11}

Patients and the public were not involved in the design of this clinical trial.

### Trial design {12}

The study is a 52-week, two-arm, parallel, double-blinded randomized clinical trial, evaluating the clinical efficacy of RAMP-Knee OA (*N* = 114) versus usual care (*N* = 114) on self-reported knee pain and other secondary outcomes. The protocol design follows the recommendation from the Osteoarthritis Research Society International [19], and the Standard Protocol Items Recommendations for Interventional Trials (SPIRIT) [20].

## Methods: participants, interventions, and outcomes

### Trial setting {13}

The study site is a university-affiliated primary care clinic in Hong Kong.

### Eligibility criteria for participants {14a}

Participants will be enrolled from eight general outpatient clinics in the New Territories East region of Hong Kong. The eligibility criteria will be screened by trained research assistants and confirmed by primary care physicians. The inclusion criteria are adults aged ≥ 45 years; a diagnosis of KOA based on the clinical criteria of the American Rheumatology College [21]; moderate-to-severe knee pain for at least 3 months, defined as a score of ≥ 4 (on a 0–10 numerical rating scale) in response to the question “What is the average level of your left/right knee pain in the past 3 months?”. Exclusion criteria include those with potentially serious knee pathology (such as inflammatory arthritis, acute trauma, or malignancy), previous knee replacement, scheduled knee joint replacement surgery within one year, and those who are unable to reliably participate, e.g., those who do not speak Chinese or unable to attend the study intervention (Table 1).

**Table 1:** Characteristics of the people who are needed for the trial

Characteristic	The people we would expect to see included
Age	Participants need to be of age 45 or above
Sex	This study recruits both male and female participants
Gender	This study recruits both men and women participants
Race, ethnicity and ancestry	No specific race, ethnicity, or ancestry requirements were set for the recruitment of this study. However, it was required that the participants could effectively communicate in Chinese (including written and spoken Chinese)
Socioeconomic status	No specific socioeconomic status requirements were set for the recruitment of this study. However, it was required that the participants could effectively communicate in Chinese (including written and spoken Chinese)
Geographic location	Since the study was conducted in a university-affiliated primary care clinic located in Shatin, Hong Kong, the participants recruited were required to be Knee OA patients in the same primary care cluster
Other characteristics relevant to the trial	*N/A*

### Eligibility criteria for sites and those delivering interventions {14b}

The study site is a primary care clinic affiliated with the Chinese University of Hong Kong. The primary investigator of this research is an associate professor at the Chinese University of Hong Kong. The study is conducted by a research nurse and research assistants employed and trained under the Chinese University of Hong Kong.

### Who will take informed consent? {32a}

Informed consent is taken by a trained research assistant.

### Additional consent provisions for collection and use of participant data and biological specimens {32b}

No additional consent is required for the collection and use of participant data and biological specimens.

## Intervention and comparator

### Intervention and comparator description {15a}

#### Intervention Group (RAMP-Knee OA)

Patients allocated to the intervention group will continue their “usual care” at the GOPCs, plus enrolment into the RAMP-Knee OA program. The intervention is designed according to the template for intervention description and replication (TIDieR) Checklist (Appendix 1) [22] and summarized in Appendix 2.

##### Name: RAMP-Knee OA

Why: The Hong Kong Reference Framework for Common Musculoskeletal Problems in Primary Care followed the recommendations of the Osteoarthritis Research Society International (OARSI) guidelines 2019, which emphasizes arthritis education, structured exercise, and weight loss as core treatment for knee OA [23]. However, clear clinical pathways and support networks are lacking in primary care to facilitate the implementation of these recommendations. The proposed RAMP-Knee OA care model will incorporate risk-stratified care planning, patient education, self-management support, and scheduled health assessments to address this evidence-to-practice gap.

What: RAMP-knee OA is designed based on the validated framework of CCM, which identifies six important components including self-management support, clinical information systems, delivery system redesign, decision support, health care organization, and community resources [24, 25]. A systematic review found that to achieve positive outcomes, at least two of these components will need to be included in interventions [24]. In this RAMP-Knee OA, 4 components will be included, i.e., self-management support, delivery system redesign, community resources, and decision support. We will also pilot the set-up of a clinical registry for patients with knee OA for potential application in the clinical information system.

We follow the treatment algorithm from the OARSI 2019 [23]. Initial assessments include confirming the diagnosis of knee OA, identifying clinically relevant comorbidities, and establishing goals and expectations. This will be followed by biopsychosocial risk assessments: physical status will be assessed by body-mass index (BMI), self-reported knee pain, subjective and objective knee function, lower limbs muscle mass, and physical activity level; psychosocial status will be assessed by participants’ mood, social network, and sleep quality. After the risk assessment, data will be entered into a registry, and individual reports will be created to identify needs and preferences (Appendix 3).

The 3 core components of the RAMP-knee OA will be arthritis education, base exercise, and weight management. The other 2 add-on components of counseling support and dietary advice on musculoskeletal health will be offered according to individuals’ specific risk factors:

(i) Arthritis education: The arthritis resources will be complied by the research team based on existing online resources. Topics will covered the 21 key messages identified in International Consensus List of Essential Statements for Osteoarthritis “What Do People With Knee or Hip Osteoarthritis Need to Know” [26].
(ii) Structured land-based exercise: The PA has established a physiotherapist-developed exercise library in a Digital Apps to facilitate exercise prescription. The exercise components have been established based on the Consensus Guidelines from the Osteoarthritis Research Society International (OARSI) Rehabilitation Group 2023 [27]. These guidelines recommend various exercises, including aerobic, strengthening, neuromuscular training, flexibility training, and balance training. We have selected strengthening, flexibility training (stretching), and aerobic exercise as our key components because they are most applicable in primary care settings.
  Regarding strength training, traditional quadriceps training typically involves knee extension exercises that may not activate the vastus medialis oblique (VMO) muscle effectively. As reduced motor recruitment is common in knee OA [28], and our previous research has emphasized the importance of VMO training [29], we have specified VMO training in our program. We appreciate your comment about core muscle training of transverse abdominis. Although core muscle training has demonstrated efficacy in young adults with knee pain and injuries, its role in knee OA is weak. Therefore, we have decided to remove core muscle training from our exercise protocol. Evidence-based training for the quadriceps femoris [30], gluteus maximus, and medius muscles will be included [31].
  For flexibility training, the hamstrings are two-joint muscles that act as flexors at the knee and extensors at the hip. Study has shown that patients with knee OA exhibit increased hamstring muscle activation, which can interfere with normal load distribution in the knee and contribute to disease progression [32]. Patients with knee OA often experience posterior knee pain due to hamstring tightness. Thus, hamstring stretching is typically prescribed to address this issue instead of strengthening. A small trial has further confirmed the benefits of combining hamstring stretching with strength training for improving knee pain, function, and mobility [33].
  The nature and dosage of exercise in our intervention align with the recommendations provided by the American College of Sports Medicine (ACSM) for aerobic fitness, muscular strength, and flexibility in apparently healthy adults. The exercise program will be individually adjusted by our nurses based on each patient’s fitness level and capabilities (Appendix 3).
(iii) Weight Management: If the participant has a BMI ≥ 23 kg/m^2^, defined as “over-weight” in Asians, they will be offered counselling support on weight management. According to the OARSI 2014, the goal will be weight loss of 5% within a 20-week period, i.e., 0.25% per week for the treatment to be efficacious [34]. Studies have shown that primary care-led weight management is effective and sustainable for long-term weight reduction and chronic disease control [35–37]. For the weight management package, we will refer to previous successful intervention components [35], and modified further according to local dietary habits and lifestyles. This will be achieved through guided food choices and portions, and a tailor-made strategy to increase incidental and general physical activity levels.
(iv) Counselling support for psychosocial health: counselling support will be provided for those with mild to moderate anxiety and depression, insomnia and loneliness identified in the risk assessment. For those with severe anxiety and depression, counselling support will still be provided if participants are already under clinical care and confirmed to be suitable to join the program by the referring PCPs. Counselling materials are available online. Community resources on psychosocial support will also be provided if necessary.
(v) Dietary advice: Age-related muscle loss impairs strength and power and can lead to a decreased ability to stabilize joints, resulting in joint misalignment, excessive joint pressure and subsequently lead to OA. Current protein recommendations by the World Health Organization (WHO) are 0.8 g protein/kg for adults and increased to 1.0–1.2 g protein/kg for people age 65 or above [38]. Sufficient vitamin D is also encouraged for overall muscle health [39]. Online dietary education for musculoskeletal health, with reference to local context, is available under the PA’s existing service.

Who provided: PCPs will be responsible to confirm the diagnosis of knee OA, identify any relevant comorbidities and optimize their control, review current use of pain medications and to refer eligible patients to the RAMP-OA program. The RAMP-Knee OA will be led by a full-time registered nurse (RN) trained by the research team. He or she will be responsible to deliver the core treatments, provide ongoing lifestyle counselling, prioritize participants’ needs, set and agree on goals, and generate strategies to meet the goals. A part-time physiotherapist and dietitian will be consulted on the design and progress of individuals’ weight management plan.

When and How: Patients will be recruited by PCPs in their usual clinic appointments, which are mostly scheduled every 4 months. Eligible patients assigned to the intervention group will be referred to RAMP-Knee OA clinic operated by RN, which will be a face-to-face consultation scheduled every 4 months following patients’ usual clinic appointments. A total of 4 sessions will be provided for each enrolled participant over one year. The first session will last for 40 minutes and serve as an introduction session; the RN will go through individuals’ biopsychosocial risk parameters, identify their needs, provide core treatment recommendations, set goals, and formulate strategies according to patients’ understanding and preferences. The second and the third sessions, each will last for 20 minutes, will provide an opportunity to review compliance with core recommendations, clarify uncertainties, provide ongoing counselling support, and monitor disease progress. The fourth session, which will also last for 40 minutes, will serve as a wrap-up session in which participants will learn about their progress over one year and reinforce life-long lifestyle modifications for healthy joint.

The RN will provide updates to the PCPs on the progress of their referred patients via electronic reminders in the Clinical Management System (CMS) after each assessment session—or upon request by the PCPs. The reports will detail any strategies and actions agreed with the patients, and their progress to date. The RN will refer the patients back if they require a medication review, e.g., if patients develop side-effects after the use of pain-killers, or if they have any other medical condition that affects participation in the trial or an escalation of care. If the situation is urgent, the RN will discuss with PA on arranging an urgent medical consultation. On the other hand, if the situation is routine, the nurse will make correspondence to the electronic message on the CMS and advise the patient to report this to their PCPs during their usual clinic appointments.

Physiotherapist and dietitian will offer structured training to our RN on weight management. They will also provide on-going support and guidance on the design of individuals’ weight management plan.

## Control group (usual care)

Patients allocated to the usual care arm will continue receiving their “usual care” at the GOPCs without any additional intervention. “Usual care” typically refers to the established and commonly provided treatments, interventions, and practices that patients would receive in routine clinical practice for their particular condition or disease. In the context of GOPCs in Hong Kong, the follow-up period for all chronic diseases, including knee OA, is typically every 4 months. Each consultation during these visits has an average duration of 3–5 minutes. Within the time constraints of these consultations, physicians will address all chronic diseases, including knee OA, whether it is the main concern or a comorbid condition. Physicians may provide brief healthcare advice, prescribe chronic medications and analgesics if necessary, and refer patients to physiotherapy if indicated. The management approach for knee OA and other chronic diseases will be solely at the discretion of the attending physicians.

### Criteria for discontinuing or modifying allocated intervention/comparator {15b}

According to the human research ethics approval, participants may withdraw from the study at any time. Participants are informed that the Principal Investigator may terminate the study and their participation at any time if they are deemed unsuitable for participation. Such termination will not affect their usual care received. A “stopping rule” will be applied to participants who underwent knee surgery or experienced severe knee pain during the study period [40]. For any emergencies during the study, participants were encouraged to go to the Accident and Emergency department for timely care. A&E visits are documented as adverse events of the study.

### Strategies to improve adherence to intervention/comparator {15c}

Participants are reminded to attend the scheduled study visits via text messages and phone calls. For participants in the intervention group, booklets were provided to record daily exercise and dietary intake as needed.

### Concomitant care permitted or prohibited during the trial {15d}

Since both intervention and control groups will continue usual care throughout the study period, referral to physiotherapy and the prescription of analgesics will be allowed. Electronic medical records should be able to retrieve the information. Other treatments such as acupuncture, herbal medicines, and over-the-counter drugs will be discouraged but allowed. Tracking of co-interventions will be done by self-reported diary and the electronic medical record. All participants will be asked to avoid intra-articular knee injection therapies during the study period. The use of co-interventions will be controlled as confounders in our analysis.

### Ancillary and post-trial care {34}

Ancillary and post-trial care is not needed for the current research study.

### Outcomes {16}

Demographic data such as age, gender, BMI, and duration of knee pain will be collected. The number and type of co-morbid illnesses will be recorded.

All outcome measures will be assessed at baseline, 16, 32, and 52 weeks. The primary outcome will be the Western Ontario and McMaster Universities Osteoarthritis Index (WOMAC; 5-point Likert) pain at 52 weeks. WOMAC is well-known to be the “gold standard” self-reported measure in KOA trials [19, 41]; a validated Chinese version of the WOMAC, with good internal consistency (Cronbach’s alpha > 0.7) and test–retest reliability (intraclass correlation coefficients > 0.80), will be used in this study [42].

Secondary outcomes include a set of biopsychosocial parameters: Physical function will be measured subjectively by WOMAC function subscale [42] and objectively by the 30-second chair and stand performance test [42, 43]. Lower limbs muscle mass will be measured by bioimpedance analysis [44]. Physical activity level will be measured by the Chinese International Physical Activity Questionnaire (Short form) [45]. Self-management efficacy will be measured by Pain-Self Efficacy questionnaire [46]. Generalized Anxiety Disorder-7 (GAD-7) and Patient Health Questionnaire-9 (PHQ-9) will be used to measure anxiety and depression, respectively [47, 48]. Insomnia will be measured by the 7-item Insomnia Severity Ind ex (ISI) [49], and loneliness will be measured by the 6-item De Jong Gierveld Loneliness Scale [50, 51]. EuroQuol-5D-5L (EQ-5D-5L) questionnaire will be used to measure health-related quality of life [51].

Other outcomes, including analgesics prescriptions, can be accurately recorded in the electronic medical records, but their consumption, along with over-the-counter drugs, will be logged using medication logs. To maintain a comprehensive record, patients will be provided with medication diaries to note down details such as the name, dosage, frequency, and any specific instructions for each analgesic they take. During regular assessment timepoints, patients will be asked to complete questionnaires that utilize the Timeline Followback method to inquire about their medication usage in the past 14 days. This method involves presenting patients with a calendar and having them retrospectively recall their intake of analgesics over a 14-day period [20].

Cost-effectiveness: cost-effectiveness will be analyzed if the intervention is effective in reducing WOMAC pain score. Quality-adjusted life years (QALYs) of the participants will be assessed by EQ-5D-5L, which is also one of our outcomes. The EQ-5D-5L health state classification instrument has five dimensions: mobility, self-care, usual activities, pain/discomfort, and anxiety/depression. Each dimension has five levels to indicate the severity of the problem in that dimension. The Hong Kong reference value sets will be used to covert EQ-5D-5L health status to the utility weight. Individuals’ QALY will be calculated by using the area under the curve approach. Self-reported data on health service utilization in the past three months will be collected at 0 months and 12 months; this will include hospitalization (frequency and days), visits to GOPCs, specialist out-patient clinics (SOPCs), and the emergency departments. The information on health service use in the public health system will be supplemented by records in the Hospital Authority electronic medical system. The cost of this program such as costs directly related to setup, administration, clinical management, and exercise courses will be recorded. But additional costs related to evaluation and research will not be included.

### Harms {17}

There are no anticipated or potential harms to the participants.

### Participant timeline {18}

**Fig. 1:**
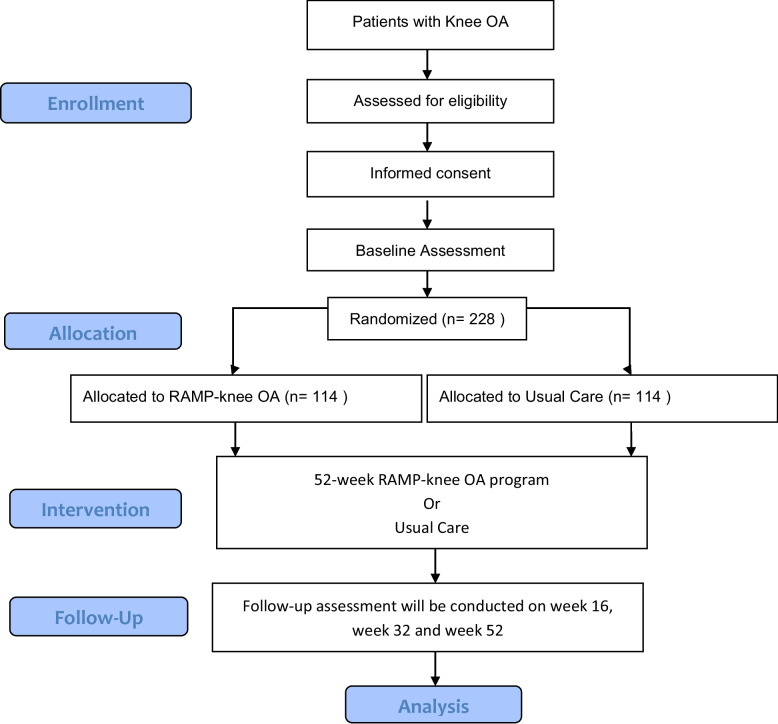
RAMP knee OA diagram

**Fig. 2:**
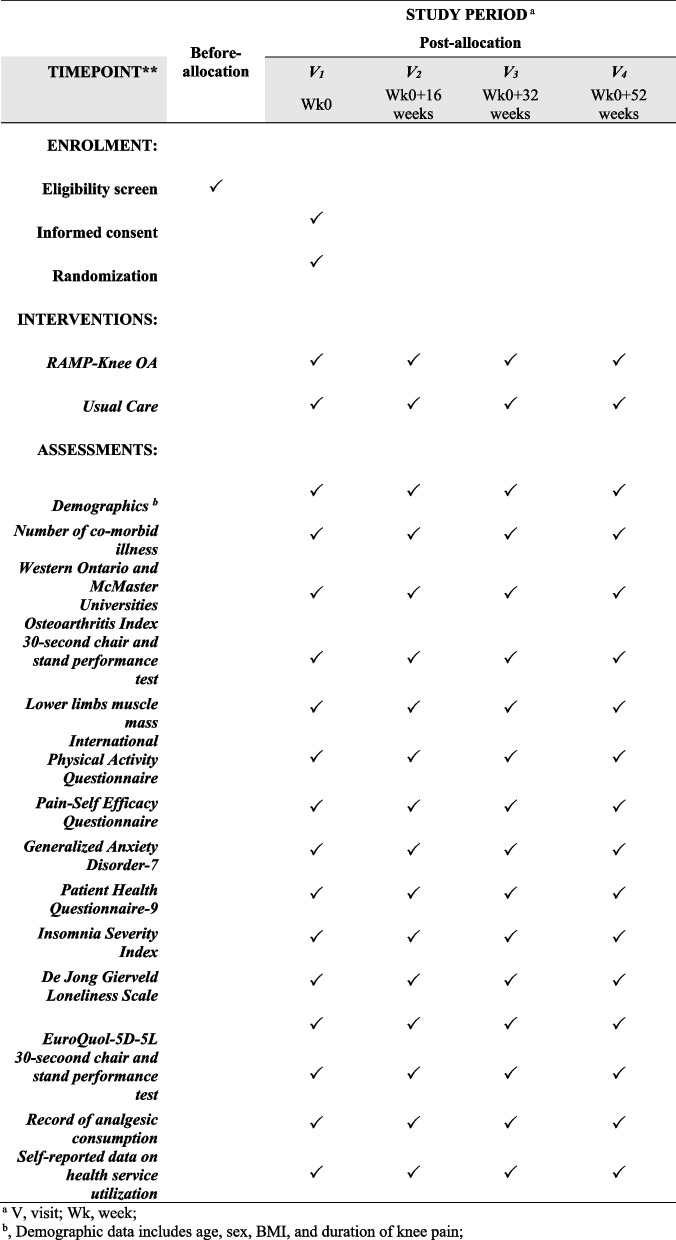
The schedule of enrolment, interventions, and assessments

Figure 1 shows the participant’s journey in this research study. Participants will first be assessed for eligibility to join. For eligible participants, informed consent will be sought. After completion of baseline assessment, participants will be randomized into one of two groups (intervention or control). Intervention group participants will attend four face-to-face sessions with the research nurse in the RAMP Knee OA intervention. Control group participants will continue with their usual care. Both groups of participants will complete study assessments at week 16, week 32, and week 52 (Fig. 2).

### Sample size {19}

There will be two arms in the study: (i) the intervention group (RAMP-Knee OA); and (ii) the control group (usual care). The sample size will be calculated based on the suggested minimal important clinical difference (MCID) of the WOMAC pain score, which is 0.469 in terms of standardized mean difference between groups [52]. Thus, a sample size of 97 in each arm will have 90% power to detect an effect size of 0.469 in a two-sample *t*-test with alpha set at 0.05 (G* power 3.1.9.7). Assuming a 15% dropout rate, the total sample size is 228, with 114 in each arm.

### Recruitment {20}

Patients will be recruited by PCPs in their usual clinic appointments, which are mostly scheduled every 4 months. Social media platforms are also utilized for the promotion of the research study.

## Assignment of interventions: randomization

### Sequence generation: who will generate the sequence {21a}

The randomization sequence is generated by a trained research associate.

### Sequence generation: type of randomisation {21b}

Randomization will be performed in a 1:1 allocation ratio [53].

### Allocation concealment mechanism {22}

The allocation sequence will be concealed through use of sequentially numbered, opaque, sealed envelopes [54].

### Implementation {23}

The allocation sequence will be concealed from the researcher enrolling and assessing participants.

## Assignment of interventions: blinding

### Who will be blinded {24a}

The research assistant and statistician will be blinded in this study.

### How will be blinding be achieved {24b}

In this open-label study, blinding of physicians, nurses, and participants will not be possible. However, all data collection will be performed by trained research assistants blinded to the allocation status of the patients, and the statistician will analyse data without referring to allocation information, thus ensuring blinding.

### Procedure for unblinding if needed {24c}

Since the participants and the research nurse are not blinded in this study, any emergency and research-related health concerns can be addressed timely and accordingly. Thus, no procedure for unbinding was put in place.

## Data collection and management

### Plans for assessment and collection of outcomes {25a}

Validated questionnaires were used to measure study outcomes. The primary outcome will be the Western Ontario and McMaster Universities Osteoarthritis Index (WOMAC; 5-point Likert) pain at 52 weeks. WOMAC is well-known to be the “gold standard” self-reported measure in KOA trials [19, 41]; a validated Chinese version of the WOMAC, with good internal consistency (Cronbach’s alpha > 0.7) and test–retest reliability (intraclass correlation coefficients > 0.80), will be used in this study [42].

Secondary outcomes include a set of biopsychosocial parameters: Physical function will be measured subjectively by WOMAC function subscale [42] and objectively by the 30-second chair and stand performance test [42, 43]. Lower limb muscle mass will be measured by bioimpedance analysis [44]. Physical activity level will be measured by the Chinese International Physical Activity Questionnaire (Short form) [45]. Self-management efficacy will be measured by Pain-Self Efficacy questionnaire [46]. Generalized Anxiety Disorder-7 (GAD-7) and Patient Health Questionnaire-9 (PHQ-9) will be used to measure anxiety and depression, respectively [47, 48]. Insomnia will be measured by the 7-item Insomnia Severity Ind ex (ISI) [49], and loneliness will be measured by the 6-item De Jong Gierveld Loneliness Scale [50, 51]. EuroQuol-5D-5L (EQ-5D-5L) questionnaire will be used to measure health-related quality of life [51].

Other outcomes, including analgesics prescriptions, can be accurately recorded in the electronic medical records, but their consumption, along with over-the-counter drugs, will be logged using medication logs. To maintain a comprehensive record, patients will be provided with medication diaries to note down details such as the name, dosage, frequency, and any specific instructions for each analgesic they take. During regular assessment timepoints, patients will be asked to complete questionnaires that utilize the Timeline Followback method to inquire about their medication usage in the past 14 days. This method involves presenting patients with a calendar and having them retrospectively recall their intake of analgesics over a 14-day period [20].

Cost-effectiveness: cost-effectiveness will be analyzed if the intervention is effective in reducing WOMAC pain score. Quality-adjusted life years (QALYs) of the participants will be assessed by EQ-5D-5L, which is also one of our outcomes. The EQ-5D-5L health state classification instrument has five dimensions: mobility, self-care, usual activities, pain/discomfort, and anxiety/depression. Each dimension has five levels to indicate the severity of the problem in that dimension. The Hong Kong reference value sets will be used to covert EQ-5D-5L health status to the utility weight. Individuals’ QALY will be calculated by using the area under the curve approach. Self-reported data on health service utilization in the past three months will be collected at 0 months and 12 months; this will include hospitalization (frequency and days), visits to GOPCs, specialist out-patient clinics (SOPCs), and the emergency departments. The information on health service use in the public health system will be supplemented by records in the Hospital Authority electronic medical system. The cost of this program such as costs directly related to setup, administration, clinical management, and exercise courses will be recorded. But additional costs related to evaluation and research will not be included.

### Plans to promote participant retention and complete follow-up {25b}

The study provides HKD$400 to each participant as a study incentive. A cash coupon of HKD $100 will be issued to each participant upon completion of each study assessment. This measure promotes participant retention.

### Data management {26}

Data will be double-entered into an electronic database.

### Confidentiality {33}

All collected personal data will be used for research purposes only. Personal data will be handled only by trained research personnel, and confidential data will be stored in an encrypted database.

## Statistical methods

### Statistical methods for primary and secondary outcomes {27a}

Baseline characteristics of the two groups will be compared using the independent samples *t*-test or Mann-Whitney test for continuous variables and chi-square test for categorical variables. Tests of intervention effects on both primary and secondary outcomes at different points will be conducted using Analysis of Covariance (ANCOVA), adjusting for baseline values. All analyses will be conducted using the R statistical program [52].

### Who will be included in each analysis {27b}

All randomized participants (i.e., intervention and control group participants) will be included in the analysis.

### How missing data will be handled in the analysis {27c}

If missing data is present, we will use linear mixed models, which is the approach to use for intention-to-treat analysis in longitudinal clinical trials with missing values [55]. Imputation of missing values before the mixed model analyses is not recommended [56].

### Methods for additional analyses (e.g., subgroup analyses) {27d}

Secondary analyses include adjustments for potential confounders, such as the use of cointerventions, and exploration of the interaction effect of treatment and the potential moderators. These will all be conducted using the R statistical program.

### Interim analyses {28b}

This is a funded study, and progress is reported to the funder at designated time points. Hence, no stopping guidelines were determined for an undesirable interim analysis result. The research assistant and statistician will have access to the interim analysis results.

### Protocol and statistical analysis plan {5}

Once published, the protocol will be available online. The de-identified data with statistical codes can be obtained from the corresponding author upon reasonable request.

## Oversight and monitoring

### Composition of the coordinating centre and trial steering committee {3d}

The study coordinating center (SCC) is composed of the principal investigator (physician), a research nurse, and the research team members. They are responsible for the conduct of the research study, discussion of study progress, and safety monitoring.

### Composition of the data monitoring committee, its role and reporting structure {28a}

A data monitoring committee that is composed of a clinician, a board-certified physiotherapist, and a data scientist is formed for data quality assurance, monitoring, and reporting.

### Frequency and plans for auditing trial conduct {29}

To monitor and ensure the quality assurance, the SCC will meet weekly to discuss the trial process and any problems encountered during the trial.

### Protocol amendments {31}

There are currently no amendments made to the study protocol. Any amendments required will be officially submitted to the Joint-CUHK-NTEC clinical research ethics committee for approval.

### Dissemination policy {8}

The results of this RCT will be disseminated through presentations at public lectures, scientific institutions, and meetings as well as through peer-reviewed scientific articles.

## Discussion

This protocol describes the rationale and implementation of the RAMP knee OA program in Hong Kong Primary Care setting. The intervention program designed is based on the validated framework and follow the OARSI 2019 treatment algorithm. It includes risk-stratified care planning, self-management support and scheduled health assessments in Hong Kong. The implementation of this study will address the 1 practice-evidence-gap for CCM in Hong Kong and provide a reference for the management plan of patients with knee OA with personalized care chronic knee OA.

## Trial status

The study protocol was approved by the Joint Chinese University of Hong Kong – New Territories East Cluster Clinical Research Ethics Committee on 5 December 2023. The identifier of the approved protocol is version 1 on 11 October 2023. Recruitment began in 1 April 2024 and completed in 12 May 2025. The last patient visit is expected on 12 May 2026.

## Supplementary Information

Supplementary Material 1.

Supplementary Material 2.

Supplementary Material 3.

Supplementary Material 4.

## Data Availability

The de-identified data with statistical codes and the relevant results in this study will be shared through academic conferences and scientific papers. The datasets can be obtained from the corresponding author upon reasonable request.

## Abbreviations

RAMP: Risk Assessment and Management Program
OA: Osteoarthritis
CCM: Chronic care model
RAMP-Knee OA: Risk Assessment and Management Program on Knee Osteoarthritis
WOMAC: Western Ontario and McMaster Universities Osteoarthritis Index
GAD-7: Generalized Anxiety Disorder-7
PHQ-9: Patient Health Questionna`ire-9
PCPs: Primary care physicians
GOPCs: General outpatient clinics
TIDieR: Template for Intervention description and replication
OARSI: Osteoarthritis Research Society International
BMI: Body mass index
ACSM: American College of Sports Medicine
WHO: World Health Organization
RN: Registered nurse
CMS: Clinical Management System
ISI: Insomnia Severity Index
EQ-5D-5L: EuroQuol-5D-5L
QALYs: Quality-adjusted life years
SOPCs: Specialist outpatient clinics
MCID: Minimal clinically important difference
ANCOVA: Analysis of covariance
SCC: Study coordinating center
