# Supplementary material for: Risk Assessment and Management Program (RAMP) on knee osteoarthritis in primary care—a one-year pragmatic randomized controlled trial
Source: Trials. 2026 Feb 3;27:187. doi: 10.1186/s13063-026-09469-x (PMC12958584; doi:10.1186/s13063-026-09469-x)
Supplement: Supplementary file 3 — Supplementary Material 3. [file 13063_2026_9469_MOESM3_ESM.docx]

Appendix 2: Digital App

| 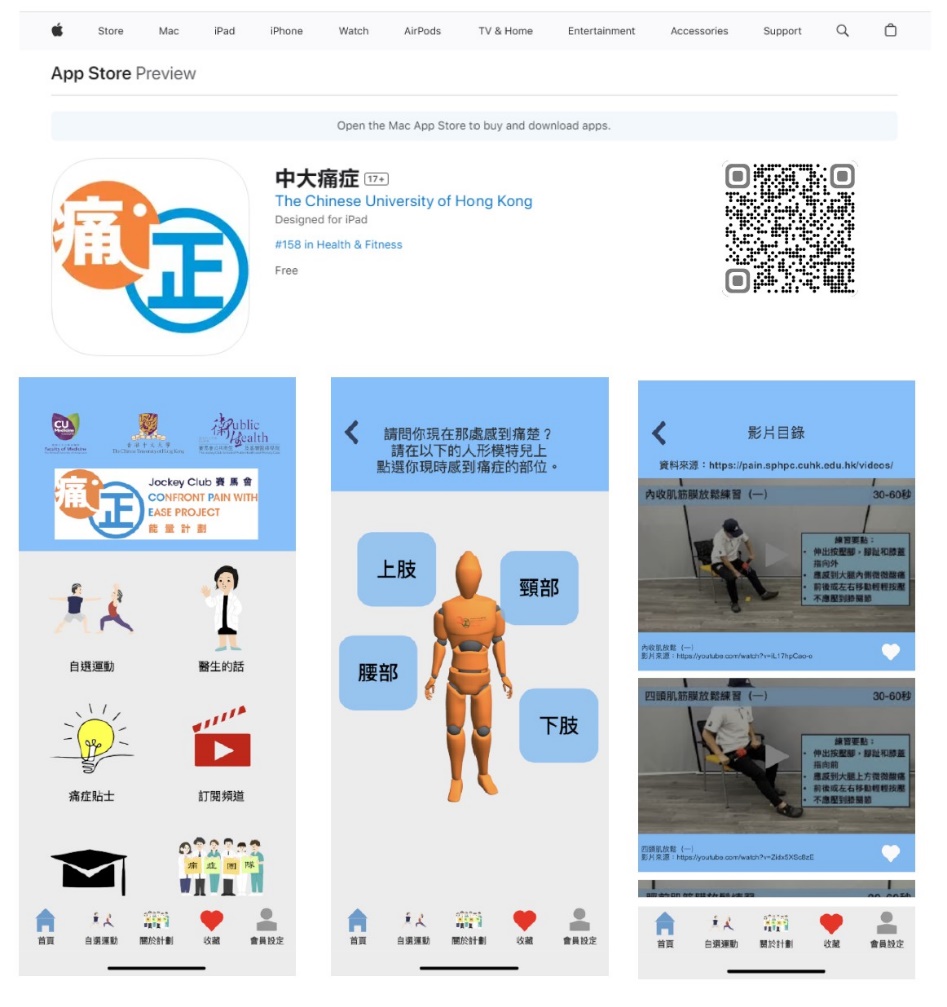 | 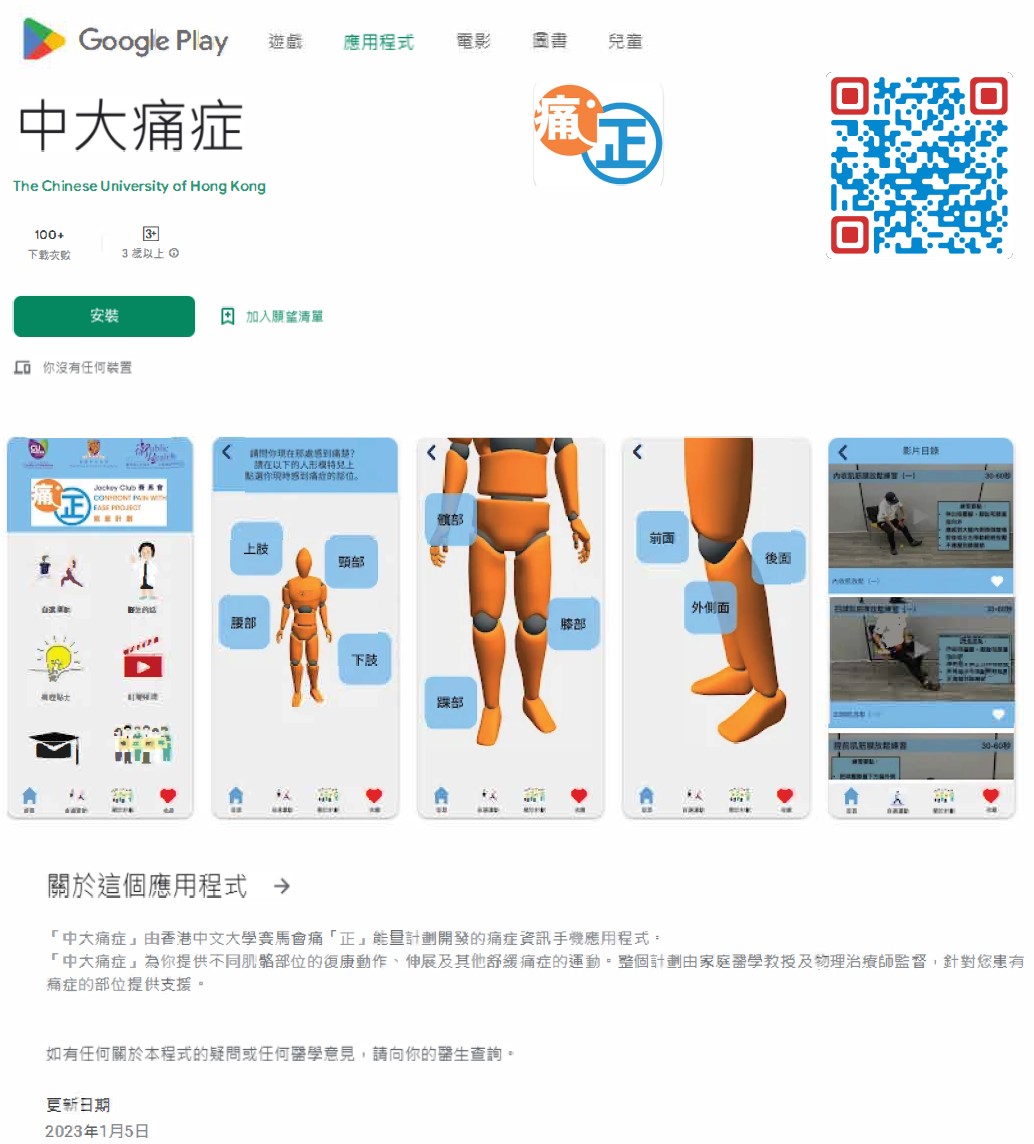 | |
| --- | --- | --- |
| Download the App from: |  | |
| Apple IOS: <https://apps.apple.com/ee/app/%E4%B8%AD%E5%A4%A7%E7%97%9B%E7%97%87/id1642857581>;  Google Play:  <https://play.google.com/store/apps/details?id=hk.edu.cuhk.sphpc.cupain&pli=1> | 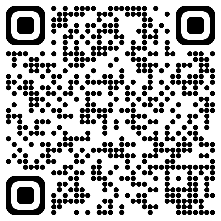  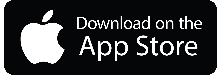 | 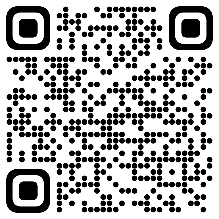  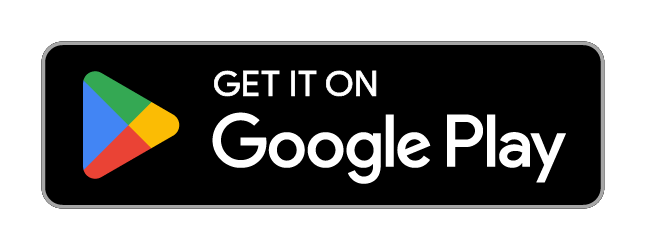 |
