# Supplementary material for: Risk Assessment and Management Program (RAMP) on knee osteoarthritis in primary care—a one-year pragmatic randomized controlled trial
Source: Trials. 2026 Feb 3;27:187. doi: 10.1186/s13063-026-09469-x (PMC12958584; doi:10.1186/s13063-026-09469-x)
Supplement: Supplementary file 4 — Supplementary Material 4. [file 13063_2026_9469_MOESM4_ESM.docx]

Appendix 3. The nature and dosage of exercise

|  | **Resistance exercise** | **Flexibility exercise (Stretching)** | **Aerobic exercise** |
| --- | --- | --- | --- |
| Target muscles | Quadricep femoris muscles, Vastus medias oblique, Gluteus maximus and medius. | Hamstring | NA |
| Intensity/workload | 55–90% of maximal heart rate | Stretch to the point of feeling tightness or slight discomfort | 55–90% of maximal heart rate |
| Duration | 8–12 repetitions or the number of repetitions needed to induce muscle fatigue but not exhaustion in 2–4 sets | 10–30 s. 2–4 reps or 60 s total stretching time | 20–90 min |
| Frequency | 2–3 days per week | ≥2–3 days per week | 3–5 days per week |
